# Garden Scraps: Agonistic Interactions between Hedgehogs and Sympatric Mammals in Urban Gardens

**DOI:** 10.3390/ani13040590

**Published:** 2023-02-08

**Authors:** Dawn Millicent Scott, Robert Fowler, Ariadna Sanglas, Bryony Anne Tolhurst

**Affiliations:** 1 School of Animal Rural and Environment Sciences, Brackenhurst Campus, Nottingham Trent University, Southwell NG25 0QF, UK; 2 School of Life Sciences, John Maynard Smith Building, University of Sussex, Brighton BN1 9QG, UK; 3 Department of Conservation Biology, Estación Biológica Doñana, CSIC, Américo Vespucio, 26, 41092 Sevilla, Spain; 4 School of Applied Sciences, University of Brighton, Brighton BN2 4GJ, UK

**Keywords:** hedgehog, *Erinaceus europaeus*, red fox, *Vulpes vulpes*, Eurasian badger, *Meles meles*, supplementary feeding, urban mammals, domestic cat, *Felis catus*, citizen science

## Abstract

**Simple Summary:**

Hedgehogs are one of several mammals that occur in urban areas in the United Kingdom and are fed by people. Food provided by people may help wild animals but may also attract animals together that could compete, injure, or predate each other. To understand the impact of food on urban animals we need to investigate how they interact when food is available. In this study, we assessed the type of interaction between hedgehogs, foxes, badgers, and cats using videos submitted by the public. We analyzed interactions between pairs of species to determine interaction type, hierarchical relationships, and the effect of food. We found that agonistic interactions (aggression and/or submission between animals) were more common than neutral interactions, and that between-species interactions showed greater ‘agonism’ than those within the same species. Of interactions within a species, those between hedgehogs were the most agonistic (54.9%) and between badgers the least (6.7%). The species interacting affected the level of agonism, with cats and foxes showing the highest level when together (76.7%). Badgers also outcompeted cats where there were contests over food, but cats were equally as successful as foxes, which were more successful than hedgehogs. However, hedgehogs dominated access to food over cats. We discuss the need to understand interactions between urban animals and the effects of providing food, to inform practice and ensure any potential risks are minimized.

**Abstract:**

Hedgehogs occur within an urban mammal guild in the United Kingdom. This guild commonly utilizes anthropogenic food provision, which is potentially beneficial to wild animal populations, but may also bring competitors and predators into proximity, raising the question of how these species interact in urban gardens. In this study, we determined interactions between hedgehogs, foxes, badgers, and domestic cats using videos submitted via citizen science. We analyzed interactions within and between species to determine interaction type, hierarchical relationships, and effect of supplementary food presence/amount. We found that overall agonistic interactions between individuals occurred more frequently (55.4%) than neutral interactions (44.6%) and that interspecific interactions showed greater agonism (55.4%) than intraspecific ones (36%). Within intraspecific interactions, those between hedgehogs were the most agonistic (54.9%) and between badgers the least (6.7%). Species composition of the interaction affected agonism, with interactions between cats and foxes showing the highest level (76.7%). In terms of overall “wins”, where access to garden resources was gained, badgers dominated cats, which were dominant or equal to foxes, which dominated hedgehogs. However, hedgehogs exhibited a greater overall proportion of wins (39.3%) relative to cats. Our findings are important in the context of the documented impact of patchy resources on urban wildlife behavior, and we show that provision of anthropogenic food can potentially result in unintended consequences. We recommend actions to reduce proximity of guild competitors in space and time to limit negative effects.

## 1. Introduction

Increasing urbanization, where habitats have been highly modified for intense human use and residence, typically has a negative effect on biodiversity [1], yet a few species appear to benefit, as evidenced by their urban colonization, and high relative densities (e.g., [2]). These species are termed “synurbic” [3]. Although urban development can present challenges for wildlife, it can also offer benefits. For example, thermal climatic conditions are more stable in urban environments, and ground and air temperatures can be higher than in surrounding rural areas [4], while towns and cities provide numerous sheltering opportunities, and abundant supplementary food from anthropogenic sources [5,6]. Among the many reasons to colonize urban landscapes, food availability is perhaps one of the most important [7].

The intentional provision of supplementary food to wild animals by urban residents, either as surplus household food or commercially purchased for this purpose, has increased in the UK [8], where an estimated 87% of urban dwellers have access to a back or front garden/yard of their residence [9]. Supplementary feeding can benefit wild populations by providing food sources when natural food sources are low. However, it can also create clustered, abundant, spatio-temporally predictable food resources in urban areas [10], leading to behavioral modifications of wildlife, such as changes in foraging and spatial behavior [6,11], diet [12], and social/territorial configuration [11,13] with corresponding changes in density. In the United Kingdom (UK), synurbic mammal species include the Western European hedgehog (*Erinaceus europaeus*) (hereafter “hedgehog”), the red fox (*Vulpes vulpes*) (hereafter “fox”), and the Eurasian badger (*Meles meles*) (hereafter “badger”). These three species form a guild within which they compete for similar food resources; however, badgers and foxes can prey on hedgehogs, i.e., intraguild predation (IGP) also occurs [14,15]. The presence of both badgers and foxes negatively predicts UK hedgehog presence [16,17], but it is unclear whether this is due to predation, competition, or a combination of both. Modeling studies suggest that intraguild predation dynamics may additionally be confounded by the presence of supplementary food [18].

Higher densities relative to rural counterparts, have been documented in hedgehogs [5,16], foxes [6], and to some extent badgers [19]. Factors affecting hedgehogs in the UK are of particular interest due to recent widespread population declines [20,21,22,23]. Within population studies, sub/urban habitats are considered potential refuges for hedgehogs [16,24,25], typically providing a range of ecologically favorable attributes, including high food availability and low overall populations of badgers, although high badger sett (burrow network) densities have been documented in some sub/urban areas [26]. This raises the question of how these species interact and co-occur in towns and cities.

Exploitation of supplementarily provisioned food in urban gardens has been observed in all three species, and although this often manifests in individuals feeding alone, co-occurrence and aggregation of multiple individuals and species can also result [8,27]. In addition to synurbic wildlife, domestic cats (*Felis catus*) occur at high densities in urban areas [28] and utilize anthropogenic food sources, spatially overlapping with urban wildlife [29]. Therefore, cats can also be considered part of the urban mammalian guild when investigating species interactions.

Most urban mammals, including cats, forage alone, despite some living in social or family groups, except for hedgehogs, which are considered solitary [30]. Hierarchy within social groups, such as those comprised of badgers or foxes, reduces competition and the risk of aggressive interactions [31]. Evidence of aggression between conspecifics is limited in hedgehogs except for in the context of mating [32]. A garden in which supplementary food is provided could be considered a ‘high-quality’ patch containing predictable resources in abundance. Individuals may compete to defend, or acquire, high-quality patches and engage in intra- and interspecific agonistic behavior (fighting or conflict behavior, such as threatening, aggressive, and/or submissive behaviors) to dominate access for their own benefit [33]. Each dyadic encounter (paired encounter between individuals) represents the balance of the possible fitness costs and benefits from competing with the opponent/s for access to the resource [34] and will also reflect hierarchical positioning within groups and between competing species.

Currently, there is a gap in our knowledge of how these species interact when coming into proximity at focal food resources. Aggregation of multiple individuals of several species in gardens attracted by food could potentially result in higher encounter rates between competitors and predators. Increasing interference competition, aggression, stress, and predation risk may result. There have been reported incidents of urban hedgehogs admitted to rehabilitation centers with injuries from suspected encounters with urban predators, including 2.3% of admissions attributed to injuries from dogs and cats [35]. Therefore, it is essential we understand the interactions between synurbic mammals, what factors drive interactions and access to food, and how coexistence in these habitats can be supported.

The aims of the study were to determine if the type of interaction between sympatric urban mammal species varied depending on which species were interacting, which of the co-occurring species was most likely to ‘win’ dyadic competitions for access to supplementary food, and finally, if presence and amount of food affected the type of interaction. We hypothesized that there would be lower levels of agonism within species, especially those with established hierarchies, and greater agonism between species. We hypothesized that the largest of the species (badger) would dominate and be the most successful at ‘winning’ access to food, and that increased food availability would increase levels of agonistic interactions between and within species. Understanding interactions between synurbic mammals and the effect of supplementary feeding on interactions can help inform best practice around food provision to prevent unintended costs to the species concerned.

## 2. Materials and Methods

### 2.1. Data Collection and Cleaning

Data on urban mammal interactions were obtained from the public following a national appeal for such video footage on a UK television broadcast (British Broadcasting Commission [BBC] “Springwatch” series) in May 2017. This program is part of a long-standing seasonal series on British wildlife that has approximately 2.5 million viewers. The broadcast explained the study aims and provided details of a link to a data capture site where videos and associated metadata could be submitted. The link was also available on the associated program website and the University of Brighton website. We did not provide instructions of an experimental set-up to follow, but instead called for people who had existing footage of multiple animals within their gardens to submit their existing footage. Once on the site, the public (volunteers) could upload their videos and answer a questionnaire on the location, video content, and food provision where the video was taken. The link was available between 29 May and 15 June 2017, during which time 683 files were submitted. Video analysis at feeding sites has previously been used as a method to determine relationships and interactions between sympatric species [36].

### 2.2. Data Handling

All data collected were downloaded to an Excel sheet with a linking unique ID code assigned to each video. Prior to analysis, data were removed that were not in video format or did not contain a hedgehog, fox, badger, or cat. We included videos where at least two adult individuals were visible, recorded in a UK residential garden between Jan 2010 and May 2017. Urban habitat was verified by using Google Maps for the postcode data submitted with the video. Multiple files from the same address/date were assumed to be consecutive recordings, so only one representative video was used. Of the 683 files submitted, 586 dyads (interactions between two individuals) [30] were extracted during behavioral analysis.

### 2.3. Behavioral Analysis

Interactions between species were assessed per dyad. When interactions involved three or more individuals, multiple dyads were derived. For each dyadic interaction, the following were recorded from the video analysis: interaction number (for videos with more than two dyadic interactions); duration of video (in seconds); visible food present (yes or no); duration of the interaction (in seconds); species; predominant behavior of animal; interaction type (neutral or agonistic) [36]; and outcome for both individuals in the dyad (“win” or “no win”) [31]. The amount of food left out (“high” or “low”, where “high” was two handfuls or more and “low” was a single handful or less) was also included from the questionnaire response. An ethogram was compiled containing detailed descriptions of six typical dyadic behaviors [37] during encounters (passive, submissive, avoidance, defensive, aggressive, attack; see Table 1). As the study focused on multiple species, some definitions included reference to a particular species. During the analysis, a predominant behavior was assigned to each member of the dyad and an interaction type then chosen to summarize the encounter. Behaviors that appeared to have no impact on either animal were deemed *neutral*, i.e., animals were passive towards each other, there was no defensive or aggressive behavioral change in the presence of another, and/or there was no observed agonistic behavior. Conversely, behaviors involving submission, threat, aggression, defense, or attack were classified as *agonistic* [36]. Dyadic outcomes for each animal were classified as either “win” (if one animal was seen eating the food or dominating the space close to the food during the video clip), “draw” if the two animals continued feeding or stayed in the garden together, or “unclear” if the video or observation ended before an outcome could be determined. A loss, draw, or unclear outcome was classified as “no win” for the individual in the dyad.

### 2.4. Data Analysis

To test the effect of dyad composition (i.e., species), food presence, and amount on interaction type and probability of wins, Generalized Linear Models (GLMs) were computed with binomial error structures in R Studio (RStudio, 2012) using R v3.6.1 [38] and packages MASS and lme4. Inter- and intraspecific dyads were modeled separately. For interaction type, both models contained dyad composition as the independent variable and interaction type (Neutral = 0, Agonistic = 1) as the dependent variable. For the intraspecific model, the highest neutral interaction dyad (badger-badger) was used as a reference in the model. For probability of wins, the independent variables comprised interaction type (Neutral = 0, Agonistic = 1), whether supplementary food was provided (No = 0, Yes = 1), and the amount of food (Low = 0, High = 1) and dyad composition (all the interspecific dyads that contain at least one of the target species for that specific model). The hedgehog-cat dyad was used as a reference for the interspecific model, as the highest neutral interaction dyad that was not deemed a potentially predatory interaction. The dependent variable in each case was win (eats the food = 1) or no win (lose, draw/shares food or is unclear = 0) for each test species. Finally, the reference level was switched for each dyad comparison (using the relevel function) so that all pairwise dyad comparisons were tested against each other.

Prior to applying models, proposed explanatory variables were checked for multicollinearity using Variance Inflation Factors (VIFs). If variables had VIFs greater than 3 or correlation coefficients more than 0.6 with other variables, they were excluded from models [39]. Model residuals were assessed for normality and heteroscedasticity. Omnidirectional stepwise selection was undertaken to build the best model in each case using the step function with the command direction = both. This procedure sequentially keeps or drops variables starting with the null (intercept only) model through testing the significance of each independent variable in a linear regression model.

## 3. Results

A total of 683 files were received from volunteers. The files collected dated from May 2010 to June 2017. Using the filtering method stated in the methods section 586 separate dyads were analyzed, representing both intra- and interspecific interactions. Two datasets were created with these data; the ‘all dyads’ dataset, which included all 586 separate dyads, and the ‘outcome’ dataset, which consisted of 331 interspecific dyads with wins, draws, and unclear outcomes recorded, alongside data on food presence and amount.

### 3.1. Interactions between Sympatric Species

Badger-badger dyads had the lowest level of agonistic interactions and were recorded to be neutral in 93.3% of interactions (Table 2). In comparison, fox-fox and hedgehog-hedgehog dyads exhibited neutral behavior in 63.6% and 45.1% of interactions, respectively. Hedgehog-hedgehog dyads had the highest number of intraspecific agonistic interactions (54.9%). The most frequent interspecific dyad was fox-hedgehog, with 143 separate instances of this dyad recorded (Table 2). The split between agonistic and neutral interactions for fox-hedgehog dyads was relatively even at 49% and 51%, respectively. Interspecific interactions between cats and foxes had the highest level of agonistic interactions (76.7%). Badger-cat dyads were the least recorded, being observed only eight times, but also showed a high proportion of agonistic interactions (75%). In every dyad containing a cat, the proportion of agonistic interactions was greater than neutral; however, the incidence of these was relatively low compared to other dyads (Table 2). Hedgehogs displayed higher agonistic interactions with foxes than badgers, although these comprised <50% of interactions.

### 3.2. Comparisons between Species Dyads

Statistical models revealed a greater level of agonistic behavior in all other intra- and interspecific dyads compared to badger-only dyads (Table 3). Statistically, only cat-fox dyads had higher agonistic interactions than hedgehog-hedgehog dyads. Cat-fox and badger-fox showed significantly higher agonistic interactions than fox-fox. Both badger-hedgehog and badger-fox agonistic interactions were greater than badger-badger but less than cat-fox. Badger-fox agonism was less than cat-fox but greater than fox-fox.

### 3.3. Species Dominance and Hierarchy

**Figure 1:**
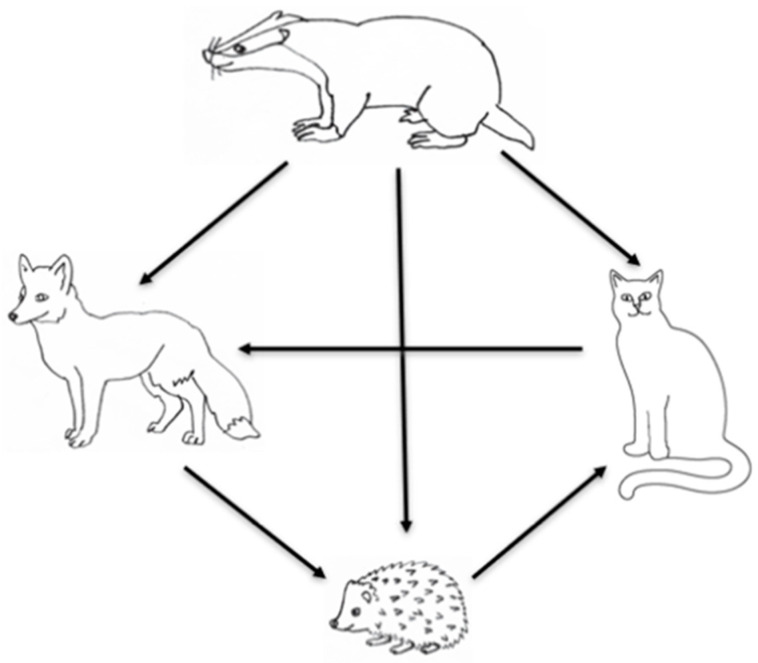
A diagrammatical representation of the hierarchical relationship in ‘winning’ access to food during paired dyads. The direction of the arrow shows the direction of the dominance is the species with the higher % of wins within dyads.

In terms of overall percentage of wins (access to garden resources), the order of dominance was firstly badgers, then cats, and then foxes. Foxes dominated hedgehogs, but hedgehogs dominated cats; hence, the hierarchy was non-linear (Table 4; Figure 1 for diagrammatical representation). However, there were a large proportion of draws and unclears in these dyads, so it is not always apparent which species won in each combination. Badgers tended to be more successful at ‘winning’ interactions than the other species, winning 45.9%, 42.1%, and 66.6% of interactions against foxes, hedgehogs, and cats, respectively. Foxes had the largest proportion of wins of any species when the dyad was with hedgehogs (46.2%). While cats won 44.4% of their dyads with foxes, foxes won 31.1% of these dyads (Table 4). Unexpectedly, hedgehogs won 39.3% of the time in dyads with cats, compared to cats winning only 10.7% of these dyads.

### 3.4. Impact of the Presence of Food

When data were applied to models to test the effect of interspecific dyad, interaction type and presence/amount of food on probability of wins, badgers won more interactions when agonistic behavior was involved in the dyad (parameter est. ± S.E. = 1.395 ± 0.413; z = 3.375; *p* < 0.001). Zero-inflated datasets prevented the inclusion of food presence and amount in some models. However, foxes exhibited a greater chance of winning when the interaction was agonistic (parameter est. ± S.E. = 2.734 ± 0.397; z = 6.878; *p* < 0.001) and food was present (parameter est. ± S.E. = 2.143 ± 0.830; z = 2.581; *p* < 0.01).

## 4. Discussion

Our study is the first to quantify behavioral interactions between hedgehogs and three other intraguild mammal species in sub/urban gardens in the UK. We have shown that food provided in gardens by human residents is utilized by a range of intraguild species with associated evidence of interactions within and between species. Based on the assessment of ‘wins’ during interactions over food access, we have derived a hierarchical relationship between a community of sympatric urban mammals. The study findings supported our hypothesis that the largest of the species (badgers) in the guild would be most successful at ‘winning’ access to food. We also found some support for our hypothesis that food availability affected interaction type, with higher levels of agonistic interactions when food was present than when it was absent, although analysis of this element was limited due to an unbalanced dataset. Spatially predictable and/or abundant food patches can create focal activity hotspots of resource exploitation, causing aggregation of sympatric species, which likely lead to higher occurrences of interactions [40]. The consequences of interactions between garden mammals are potentially numerous. Direct interactions between competing species can be aggressive, leading to injury or death, with increased competition or competitive exclusion reducing access to resources for subordinate species or individuals, with knock-on welfare effects. Furthermore, the dynamics of space use overlap and interaction rates within wild populations and between domestic and wild species has implications for pathogen transmission, particularly in urban areas at the interface between humans and wildlife, where zoonoses can emerge [41].

### 4.1. Intraspecific Interactions

Our findings support the hypothesis that agonistic behavior would be higher between species compared to within species. As expected from previous studies [31], we also showed there were few agonistic interactions between badgers at feeding sites (7%). It appears that this observation is consistent between urban and rural badger populations, despite differences in urban badger group size, density, and territory size compared to rural [19]. As expected, foxes showed higher intraspecific agonism than badgers. Although foxes typically forage alone, there is a hierarchy within fox social groups [42]. Subordinate foxes have been shown to use supplementary food patches in urban gardens in a different way to dominant foxes, using fewer patches, spending less time in predictable patches, and feeding later [43]. This is explained as an evolved behavioral strategy to reduce competition/antagonism within social groups.

Hedgehogs are considered solitary and non-territorial [32]. Field studies of behavior have shown that hedgehogs tend to avoid each other, and adults are usually only found together during courtship, or when attracted to a localized food source. Other than during courtship, when females are typically aggressive towards males, and competing male suitors may fight each other, overt aggression is rarely seen [44]. Contrastingly, we observed high levels of agonism between wild hedgehogs. This included a characteristic behavior where one hedgehog attacked another by running at it, causing the victim to roll up, after which the attacker pushed it away. Typically, the function of this behavior appeared to involve moving a competitor away from the food source, such as to the edge of the garden. In one case, an individual was pushed down a flight of concrete, and another into water. We termed this behavior ‘barge and roll’ and deemed it to be competitive.

Many of these observations of agonism between hedgehogs occurred outside of the breeding period. Anecdotal evidence from rescue centers suggests aggression can occur when food is provisioned to a group; thus, the most likely explanation for agonism in our study is defense of food patches. This behavioral disparity may be a consequence of patchy distribution and abundance of urban food resources relative to rural or wilderness areas. Previous studies on red deer (*Cervus elaphus*) have shown increased aggression at food patches when provided with spatially and temporally predictable supplementary food during winter [45]. Access to food that will allow for an increase in body weight may be more critical for hibernating species such as hedgehogs than species that are active all year, as very low body weight could affect overwinter survival [46,47]. As the focus of our study was to investigate intraguild interactions of urban wildlife, we did not request videos of cat-cat interactions in this study, and thus, could not determine such interactions at garden feeding sites.

### 4.2. Interspecific Interactions and Hierarchy

Between-species interactions were more agonistic overall than those within species, and badgers tended to be more successful at securing food than all other species. In our study, badgers were dominant over foxes where contests occurred, but if initial interactions did not escalate to aggression, each species was unaffected by the presence, proximity, or orientation of the other (consistent with [34]). Nonetheless, our study is a temporal snapshot, and previous experience may affect what was observed [36]. Interspecific interactions involving hedgehogs showed the highest levels of agonism with cats, although cats were predominantly observed to be submissive during these interactions, allowing hedgehogs access to food without contest. Hedgehogs co-occurred in urban gardens with potential predators (badgers and foxes) and approximately half of encounters between hedgehogs and foxes were agonistic. Hedgehogs have previously been reported to avoid predators [48], but in urban gardens where food is provided, they may not always do so, with the benefits of access to food provision potentially outweighing predation risk.

As cats are typically fed by owners, access to additional food is less critical to their survival or fitness compared to wild counterparts, although they are highly territorial [49] and likely to defend the gardens within their territories. We observed high levels of agonism between cats and foxes, with the former appearing dominant over the latter overall. The relatively high proportion of hedgehog wins over cats was unexpected but may relate to hedgehog spines, which domestic cats are not physically or behaviorally adapted to defend themselves against, as compared to wild predators. Although competition could impact access to food, previous studies have shown that there is more food available in urban areas than some species metabolically require (e.g., foxes [12,42,43]), which suggests that if an animal unsuccessfully forages at one patch, there are likely to be multiple alternative patches where competition could be lower due to temporal activity partitioning [43]. These may be readily accessible to more mobile species, such as foxes [50], whereas hedgehog movement can be affected by barriers in urban areas [51]. This may account for the higher-than-expected agonism observed between hedgehogs, i.e., if the value of a feeding patch is perceived to be high and movement between patches is restricted, defense may be adaptive.

Hierarchical positioning of the garden mammal guild is dominated by badgers. Foxes and cats then rank approximately equally, with cats dominating access to food over foxes, and hedgehogs dominating food over cats. However, just under half of contest outcomes overall were either unclear or a draw, and the same proportion involved no contest at all, i.e., neutral co-occurrence. Thus, “noise” in the dataset was substantial.

### 4.3. Disease Transmission Risk

Increases in space use overlap can increase both intra- and interspecific pathogen transmission risk [41]. We showed evidence of animals coming into proximity and utilizing similar feeding sites within urban gardens and interactions between and within species, as well as between wild and domestic animals. Common pathogens spread by the species in this study include the sarcoptic mange mite (*Sarcoptes scabiei*) [52] and the dog roundworm (*Toxocara canis*) [53]. Rabies lyssavirus [54] and *Echinococcus multilocularis* [53,55] are transmitted between foxes, cats, and badgers in Europe, and have the potential to emerge in the UK, although *E. multilocularis* has not yet been detected in European hedgehogs [56]. Food provision practice that reduces spatio-temporal species overlap could, thus, also reduce pathogen transmission.

### 4.4. Application of Findings to Hedgehog Conservation

This study has important implications for hedgehog conservation, in the context of both intraguild predation, and competition. Previous studies report hedgehog abundance to be negatively associated with badger abundance [57], with experimental evidence showing a doubling of hedgehog populations when badgers were absent or present at very low density, i.e., meso-predator release [58]. In our study, badgers and hedgehogs co-occurred at feeding sites, although most interactions between them were classed as neutral, including occasions where both species ate simultaneously close together. Where agonistic interactions did occur, approximately 2/3rd of these were seen as competitive, with 1/3rd considered potentially predatory, representing a small proportion of overall interactions involving these two species (10%). Although we did not record any direct hedgehog predation events by foxes in our study, 5% of agonistic interactions between the two species were classed as potential predatory behavior, with the remaining agonistic interactions considered competitive. Levels of fox-hedgehog agonism were, thus, greater than badger-hedgehog agonism, but in the form of competition rather than predation. Research conducted in Regents Park in London, UK, found foxes to be a major cause of hedgehog mortality due to injury, although this may also arise from competitive interactions rather than direct predation [59]. Rehabilitation centers report occasional admission of hedgehogs with injuries from wild predators, but these may also be caused by domestic pets [35]. Any agonistic interaction could potentially result in injury or death, and mechanisms to prevent co-occurrence at feeding sites are likely to reduce both outcomes.

However, feeding sources are not always utilized by multiple animals simultaneously, with solitary feeding regularly observed. In our study, feeding did not always lead to interactions and many co-occurrences were neutral. Supplementary feeding can benefit hedgehogs by providing food at times when natural food resources are low, potentially contributing to increased abundance [5]. Nonetheless, it is pertinent to adopt strategies to minimize negative effects of agonism towards hedgehogs according to the precautionary principle. Variable food availability in space or time, for example, reduces its predictability and the corresponding spatio-temporal overlap between or among species. Strategies to do so could include altering timing of food provision and providing food in locations with restricted (species-specific) access, such as hedgehog feeding boxes (although this would not reduce intraspecific hedgehog agonism) or at multiple sites within the garden.

As urban areas are considered refuges for hedgehogs [23], understanding how to minimize negative impacts of interactions in urban habitats will aid conservation management. In general, hedgehogs do not commonly inflict injury on conspecifics [35], although injury/other consequences of the frequently observed ‘barge and roll’ behavior are currently unknown. High levels of agonism and the associated risk of lethal/sublethal effects can be disproportionately high where hedgehogs aggregate around feeding sites in urban gardens. Sublethal effects such as exacerbated health conditions and reduced fecundity and breeding success [60] may follow competitive exclusion from food resources and associated stress. It is, therefore, recommended that feeding practices also aim to reduce hedgehog intraspecific agonism.

### 4.5. Use of Citizen Science and Data Limitations

Citizen science data collected from volunteers can elucidate species interactions in inaccessible areas, such as urban gardens [61]. Collecting data in private gardens can be intrusive to owners, involve a lot of time, and be logistically challenging [62]. To achieve a sufficiently large sample size of independent videos throughout UK sub/urban areas would be challenging in a short time frame; hence, national citizen science approaches were deemed preferable in our study. One limitation of this approach is lack of standardization in video collection, although standardized video processing can partially compensate. Participant responses to questions on presence and abundance of food provision were also difficult to standardize; thus, the trustworthiness of questionnaires is a limitation in their use [63]. Overall representativeness of datasets can also be compounded by the lower likelihood of citizen scientists submitting less ‘interesting’ data, which in our study could mean a bias toward dramatic interactions, potentially overestimating levels of agonism. In addition, in our study an unbalanced dataset arose from cameras typically being deployed at locations where animals were already encouraged such as feeding sites; hence, footage from sites with food absent is limited. The robustness of the model including food presence/absence and amount was, therefore, inevitably reduced relative to other analyses. Finally, grouping of the “no-win” category in video processing to include ‘draws’ and ‘unclear’ interactions as well as losses to allow computation of the binomial regression analysis, reduced our capacity to model nuance in species interactions.

## 5. Conclusions

Citizen science is a useful method of investigating urban wildlife, where access to private spaces is limited, with the main challenge being access to participants across a broad geographic range, although media recruiting can partially compensate for this. Animal interaction studies have benefited from the increasing availability, affordability, and ease of use of remote monitoring cameras to the public in recent years. Urban wildlife behavior is a suitable focus to engage the public, as species are familiar and interesting interactions can be easily observed at close quarters within private spaces.

Our study is the first to quantify interactions within a sympatric urban mammal community and document hierarchical relationships between wild and domestic mammals in urban gardens. We show that badgers tend to dominate this hierarchy and that high levels of agonism can occur between hedgehogs within sub/urban populations. We also report relatively high levels of agonism between hedgehogs and their potential predators, although the majority of these were competitive interactions, and actual predation events were rare. Clearly, clustering of supplementary food sources in urban environments through anthropogenic feeding can lead to multi-species co-occurrence (including both domestic animals and wildlife) and high species abundance at feeding sites. Where species co-occur at food patches, agonism is typically higher, with potential for increases in transmission rates of some pathogens. Distribution of food sources in urban areas affects the spatial ecology of synurbic animals, in addition to interaction dynamics within and between co-occurring species. Further research is necessary on the impact of supplementary food in the urban environment on animal health, ecology, and disease risk via changes in species interactions. As urban areas are considered hedgehog refuges, understanding interactions within the guild they occupy is critical for informing conservation and welfare management, including feeding practices, for this declining species.

## Figures and Tables

**Table 1:** Behavior categories and types of interaction based on analysis of encounters between four species of urban mammal—Western European Hedgehog (*Erinaceus europaeus*), Eurasian badger (*Meles meles*), Red fox (*Vulpes vulpes*), and domestic cat (*Felis catus*). Symbols represent each behavior and how they relate to the two broad interaction types.

Animal Behaviour		Interaction Type	
Passive 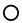	Animal continues feeding or remains in the area when approached by the other individual. Departs without retaliation if aggression occurs.	Neutral 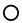 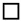 ▬	Behaviour of both animals has no impact on the other, e.g. ignore each other.
Submissive 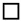	An act or posture that does not challenge the incoming animal. Body position or response indicting lower hierarchy or submission, e.g. in foxes the body and head lower. In cats crouching with ears flattened, avoiding, retreating or fleeing.		
Avoid ▬	Animal moves or backs away from the area or other animal before close proximity or physical contact. Often with body held low, casting repeated glances at the one which stays.		
Defensive █	A defensive posture or positioning e.g. piloerection, frowning or complete rolling in hedgehogs. In cats, hissing or piloerection.	Agonistic █ ► 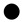 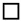 ▬	Includes submissive, threat, attack and aggression behaviour. Could result in injury, or death, of at least one individual.
Aggressive ►	Vocalisation or aggressive posture. Action of initiating physical contact with another animal including lunging, biting, scratching etc. Foxes side profile body position with arched back and head up.		
Attack 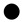	One animals runs towards the other in an aggressive manner. Chases, lunges, bites etc. In badgers, head is lowered in a threat posture and pursues the challenger with physical contact. Cat striking with paw.		

**Table 2:** The number of events recorded from videos sent in by volunteers for each dyad of four species of urban mammal (Western European Hedgehog (*Erinaceus europaeus*), Eurasian badger (*Meles meles*), Red fox (*Vulpes vulpes*), and domestic cat (*Felis catus*)) and the number and relative percentage of agonistic or neutral interactions.

	Dyad	No. of Events	No. of Agonistic Interactions N (%)	No. of Neutral Interactions N (%)
Intraspecific Dyads	Hedgehog-Hedgehog	142	78 (54.9)	64 (45.1)
	Badger-Badger	95	7 (6.7)	88 (93.3)
	Fox-Fox	33	12 (36.4)	21 (63.6)
	Total	270	97 (36.0)	173 (64.0)
Interspecific Dyads	Fox-Hedgehog	143	70 (49.0)	73 (51.0)
	Badger-Hedgehog	16	5 (31.3)	11 (68.7)
	Cat-Hedgehog	28	16 (57.1)	12 (42.9)
	Badger-Fox	78	45 (57.7)	33 (42.3)
	Fox-Cat	43	33 (76.7)	10 (23.3)
	Badger-Cat	8	6 (75.0)	2 (25.0)
	Total	316	175 (55.4)	141 (44.6)

**Table 3:** Generalized Linear Models (GLM) of the test results investigating the effects of intraspecific and interspecific dyadic composition on interaction type (Neutral = 0; Agonistic = 1), using the relevel command in R to switch the levels to investigate all dyad comparisons against each other. Displayed are parameter estimates ± standard error, z-value and *p*-value for each pairwise comparison. ‘<’ agonistic interactions are greater than the reference dyad and ‘>’ are less than the reference dyad.

Reference Dyad	Explanatory Variables	Parameter Estimate ± SE	z-Value	*p*-Value
Badger-Badger	<Cat-Hedgehog	2.819 ± 0.548	5.147	<0.001
	<Cat-Fox	3.725 ± 0.533	6.984	<0.001
	<Hedgehog-Hedgehog	2.729 ± 0.427	6.386	<0.001
	<Badger-Cat	3.63 ± 0.906	4.007	<0.001
	<Badger-Fox	2.845 ± 0.455	6.249	<0.001
	<Badger-Hedgehog	1.743 ± 0.668	2.612	<0.01
	<Fox-Fox	1.972 ± 0.534	3.692	<0.001
	<Fox-Hedgehog	2.490 ± 0.427	5.832	<0.001
Hedgehog-Hedgehog	<Cat-Fox	0.996 ± 0.398	2.5	<0.05
Fox-Fox	<Cat-Fox	1.754 ± 0.511	3.431	<0.001
	<Badger-Fox	0.870 ± 0.428	2.031	<0.05
Badger-Hedgehog	<Cat-Fox	1.982 ± 0.649	3.054	<0.01
Fox-Hedgehog	<Cat-Fox	1.236 ± 0.398	3.106	<0.01
	>Badger-Badger	−2.490 ± 0.427	−5.832	<0.001
Badger-Fox	<Cat-Fox	0.884 ± 0.428	2.067	<0.05
	>Fox-Fox	−0.870 ± 0.428	−2.031	<0.05
Cat-Fox	>Hedgehog-Hedgehog	−0.996 ± 0.398	−2.5	<0.05
	>Badger-Badger	−3.725 ± 0.533	−6.984	<0.001
	>Badger-Fox	−0.884 ± 0.428	−2.067	<0.05
	>Badger-Hedgehog	−1.982 ± 0.649	−3.054	<0.01
	>Fox-Fox	−1.754 ± 0.511	−3.431	<0.001
	>Fox-Hedgehog	−1.236 ± 0.398	−3.106	<0.01

**Table 4:** The number of events recorded from videos sent in by volunteers between each dyad of four species of urban mammal (Western European Hedgehog (*Erinaceus europaeus*), Eurasian badger (*Meles meles*), Red fox (*Vulpes vulpes*), and domestic cat (*Felis catus*)), and the number and relative percentage (%) of ‘wins’ for each species within the dyad.

		Win				Draw	Unclear
Dyad	No. of Events	Hedgehog	Badger	Fox	Cat		
Fox-Hedgehog	145	14 (9.6)		67 (46.2)		41 (28.3)	23 (15.9)
Badger-Hedgehog	19	2 (10.5)	8 (42.1)			7 (36.8)	2 (10.5)
Hedgehog-Cat	28	11 (39.3)			3 (10.7)	9 (32.1)	5 (17.9)
Badger-Fox	85		39 (45.9)	8 (9.4)		30 (35.3)	8 (9.4)
Cat-Fox	45			14 (31.1)	20 (44.4)	7 (15.6)	4 (8.9)
Badger-Cat	9		6 (66.6)		1 (11.1)	1 (11.1)	1 (11.1)
Total	331	27 (14.1)	53 (46.9)	79 (28.7)	24 (29.3)	101 (30.5)	43 (12.9)

## Data Availability

Restrictions apply to the availability of these data. The data are not publicly available due to data use restrictions stated when obtained data from the general public.
